# Supplementary material for: Health and Safety Practices as Drivers of Business Performance in Informal Street Food Economies: An Integrative Review of Global and South African Evidence
Source: Int J Environ Res Public Health. 2025 Aug 8;22(8):1239. doi: 10.3390/ijerph22081239 (PMC12386124; doi:10.3390/ijerph22081239)
Supplement: Supplementary file 1 [file ijerph-22-01239-s001.zip › ijerph-3761353-supplementary.pdf]

Supplementary Figure S1: PRISMA DIAGRAM

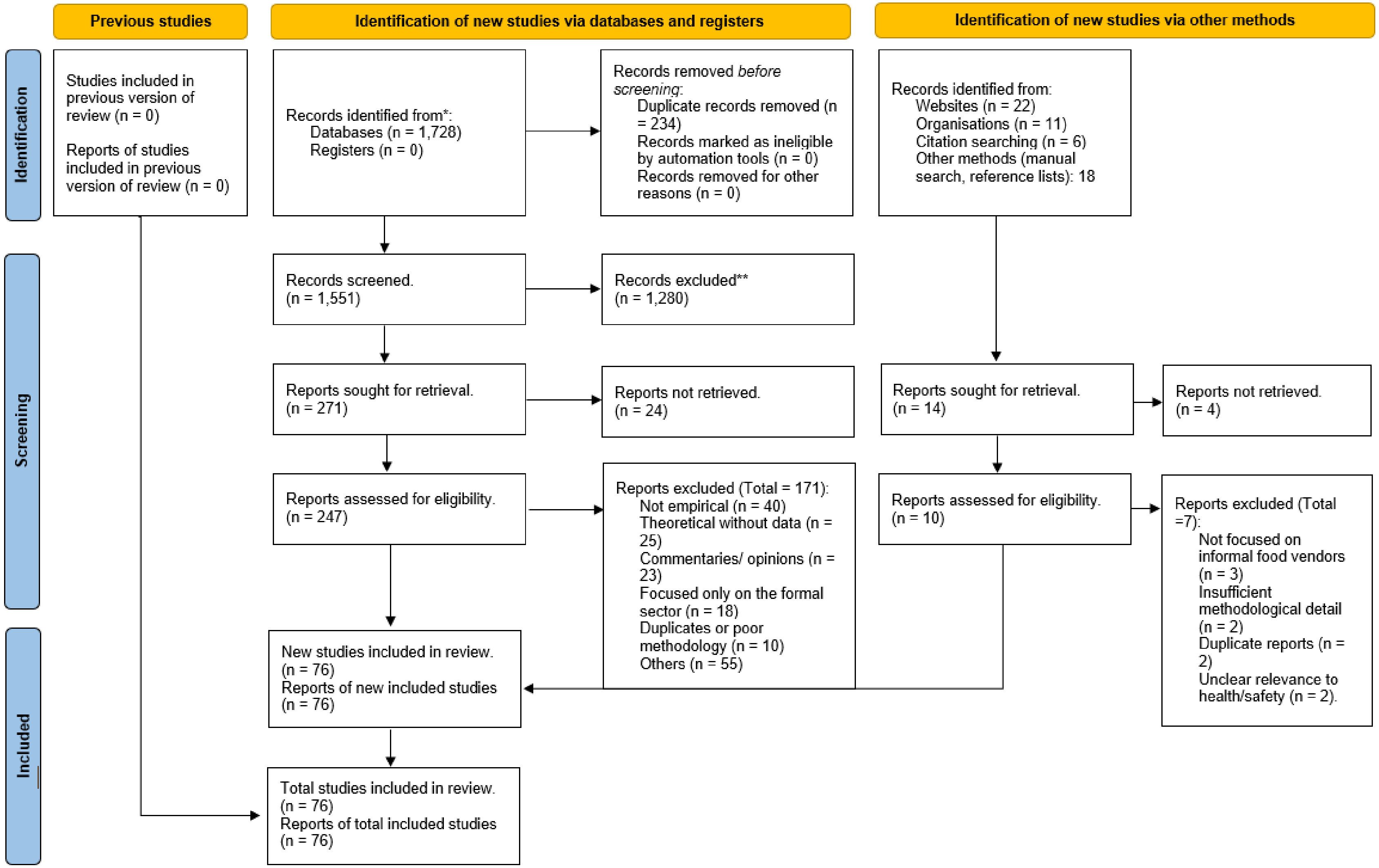

Supplementary Table S1: Data Extraction Matrix for Health and Safety Practices as Drivers of Business Performance in Informal Street Food Economies

Inclusion No	Author	Year	Title	Country	Study Type	Focus Area	Framework	Key Findings	Mapped Theme
1.	Oladipo-Adekeye, O. T.	2020	The food safety knowledge of street food vendors...	South Africa	Empirical	Food safety knowledge	None	Poor knowledge among vendors affects compliance in Johannesburg.	Vendor Knowledge
2.	Castro, M. M.	2021	Could easing restrictions on street-food vending improve public health and the economy?	USA	Commentary	Policy reform	None	A flexible policy may boost safety and public health outcomes.	Policy and Regulation barriers
3.	Masuku, B., & Nzewi, O.	2021	Socio-economic exclusion from basic service provisions...	South Africa	Empirical	Informal sector policy	None	Exclusion from infrastructure impedes compliance in Buffalo City.	Policy and Regulation barriers
4.	Mahopo et al.	2022	Operational characteristics of women street food vendors in rural South Africa	South Africa	Quantitative cross-sectional	Vendor operations, gendered dynamics, food safety practices	None	Women vendors in rural Limpopo often operate informally, face infrastructure deficits (water, sanitation), and have limited access to hygiene training. Despite this, many adopt informal safety routines. Licensing is rare, and income is modest but crucial for household survival.	Gendered vulnerabilities; Barriers to compliance; Health and livelihoods intersection
5.	Desye, B. et al.	2023	Food safety knowledge, attitude, and practice...	Multiple LMICs	Systematic Review	KAP among vendors	None	Gaps in knowledge and practice across LMICs.	Vendor Knowledge
6.	Hilmi, M.	2020	Street food vendors' marketing characteristics in 12 countries	Global	Empirical	Entrepreneurial practices	None	Vendors lack structured hygiene-oriented marketing strategies.	Business Practice
7.	WHO	2022	Food safety is everyone's business in traditional food market	Global	Guideline	Hygiene best practices	None	Outlines foundational hygiene behaviours.	Food Safety Education
8.	Nkosi, N. V., & Tabit, F. T.	2021	Food safety knowledge of vendors and sanitary conditions	South Africa	Empirical	Sanitary conditions	None	Zululand vendors show inconsistent sanitation and knowledge.	Vendor Hygiene
9.	Yakubu, M., et al.	2023	Competence of street vendors to provide nutritious and safe food	Ghana	Survey	Nutrition and food safety	None	Knowledge varies; structured training is needed.	Vendor Training
10.	Sepadi & Nkosi	2023	Health Risk Assessment of Informal Food Vendors	South Africa	Comparative Study	Health risks	HBM	Identifies risk pathways and differences in compliance across zones.	Risk Assessment
11.	Abdullah et al.	2020	Informal control and safety measures of street hawkers	Ghana	Narrative Study	Informal controls	None	Vendors rely on peer norms and informal networks for hygiene practices.	Informal Regulation
12.	Zeb, S., Hussain, S. S., & Javed, A.	2021	COVID-19 and a way forward for restaurants and street food vendors	Pakistan	Conceptual/Policy	Post-COVID adaptation	None	Explores the economic and operational impact of COVID-19 on informal food vendors; proposes strategic recovery plans.	Policy and Regulation barriers; Public health disruption and business adaptation
13.	Hill, J., et al.	2019	Food sold by street food vendors in Cape Town and surrounding areas	South Africa	Empirical	Food prep and nutrition	None	Vendors lack comprehensive food and nutrition knowledge, affecting safety.	Vendor Knowledge
14.	Khuluse, D. S., & Deen, A.	2020	Hygiene and Safety Practices of Food Vendors	South Africa	Empirical	Hygiene practices	None	The majority of vendors lack proper hygiene infrastructure.	Hygiene Compliance

15.	Hariparsad, S., & Naidoo, R. N.	2019	Effects of occupational pollutants on reproductive health	South Africa	Cross-sectional	Occupational health	HBM	Informal vendors face reproductive health risks due to exposure.	Health and Safety Risk
16.	Ramos, A. K., et al.	2021	Health of meatpacking workers using HBM	USA	Empirical	Occupational safety	HBM	Shows practical HBM application for occupational health.	Theory Application
17.	Sepadi & Nkosi	2023	Strengthening Urban Informal Trading and Improving the Health of Vendors	South Africa	Empirical	Health of informal vendors	Integrated	Offers a model linking health improvement to informal sector management.	Policy Integration
18.	Gamieldien & van Niekerk	2017	Street vending in South Africa: An entrepreneurial occupation	South Africa	Theoretical/Empirical	Economic livelihood	None	Frames vending as entrepreneurship, requiring municipal support.	Socioeconomic Lens
19.	Petersen & Charman	2017	Scope and scale of informal food economy in South African townships	South Africa	Empirical Census	Business scale and operations	None	Maps, scale, and growth of informal food enterprises.	Economic Mapping
20.	Sepadi, Dyantyi & Nkwana	2025	The Potential of Artificial Intelligence in Enhancing Occupational Health and Safety	South Africa	Conceptual Case Study	Technology & OHS	AI	Discusses the AI potential for improving OHS among informal vendors.	Innovation in OHS
21.	Gbaffou, A. B.	2016	Do street traders have the ‘right to the city’?	South Africa	Political Analysis	Rights of vendors	Urban Justice	Explores trader organizations' role in defending informal vendors’ rights post-‘clean sweep’ operations.	Governance & Inclusion
22.	Lagardien, Cousins & Muanda	2015	Community-Led Total Sanitation in South Africa	South Africa	Policy Guide	Sanitation strategies	CLTS	Provides tools to support grassroots sanitation and hygiene in informal settings.	Sanitation Approaches
23.	Zogli, Dladla & Dlamini	2021	Assessing the challenges faced by informal street traders in Durban	South Africa	Empirical	Challenges in the informal economy	None	Finds vendors face significant operational and regulatory barriers in urban settings.	Urban Informality
24.	Mjoka, Selepe & du Preez	2016	Business practices of selected street food vendors in KwaDlangezwa	South Africa	Empirical	Business practices	None	Investigate hygiene and business practices in KwaZulu-Natal vendors.	Hygiene & Operations
25.	Knox et al.	2019	Aspirations to grow: Informal enterprises in the street food sector	South Africa	Qualitative Study	Micro-enterprise aspirations	None	Vendors express strong growth intent but lack structural support.	Business Development
26.	Klutse & Sampson	2025	Assessment of hygiene practices and knowledge of food safety among street vendors in Volta Region	Ghana	Empirical	Hygiene and safety	None	Most vendors had inadequate hygiene practices despite moderate knowledge.	Hygiene Compliance
27.	Addo-Tham et al.	2020	Food safety and handling practices in Ejisu-Juaben Municipality	Ghana	Cross-Sectional Study	Handling practices	None	Shows a weak link between hygiene knowledge and actual practice.	Practice Gaps
28.	Dundery & Addo	2016	Food hygiene awareness and practice among vendors in Ghana	Ghana	Quantitative survey	Awareness & hygiene	None	Emphasizes poor sanitation infrastructure hinders good hygiene even among aware vendors.	Infrastructure Barrier
29.	Elsahoryi et al.	2024	Food safety knowledge, attitudes and practices of street vendors: A cross-sectional study in Jordan	Jordan	Cross-sectional	KAP on food safety	None	Revealed significant gaps between knowledge and practice despite high awareness.	Compliance & Practice Gap
30.	Adebayo O. O and Oyetola, B. T	2021	Food Safety and Hygienic Practices Among Food Vendors in Egbeda Local Government Area, Ibadan Oyo State	Nigeria	Survey	Food safety KAP	None	Demonstrates high knowledge but poor hygiene facilities impact safety practices.	Infrastructure Barrier

31.	Bautista-Bernal et al.	2024	Safety culture, safety performance, and financial performance: A longitudinal study	Not Specified	Longitudinal Study	Safety & business outcomes	Safety Culture	Links robust safety culture to improved financial outcomes in food enterprises.	Safety & Profitability
32.	Huynh-Van et al.	2022	Factors associated with food safety compliance among street food vendors in Can Tho City, Vietnam	Vietnam	Mixed-methods	Compliance factors	None	Identifies training and experience as key predictors of hygiene compliance.	Training & Compliance
33.	Irawati, Rustono & Farouk	2019	Effects of occupational health and safety culture on employees' performance at Agility International Ltd.	Indonesia	Case Study	OHS culture	None	Positive safety culture correlates with improved staff performance.	OHS Impact
34.	Liguori	2021	How do food safety concerns affect consumer behaviors and diets in LMICs?	Global	Systematic Review	Consumer food safety behavior	None	Concerns about food safety significantly affect dietary choices in LMICs.	Consumer Perception
35.	April et al.	2020	Consumer motivations and barriers to alternative food purchase in Ecuador	Ecuador	Cross-sectional	Consumer preferences	None	Reveals that trust and perceived hygiene are major barriers to street food adoption.	Consumer Trust
36.	Gupta et al.	2018	Street food dimensions and its effects on consumer attitudes and behavioral intentions	India	Survey	Consumer behavior	None	Consumers value hygiene and presentation; it impacts intention to purchase.	Hygiene & Customer Intentions
37.	Nortey et al.	2024	Knowledge, attitude, and food safety practices among street food vendors in Ghana	Ghana	Cross-sectional	Food safety KAP	None	Finds a correlation between formal training and improved hygiene practice.	Training Effectiveness
38.	Tuglo et al.	2021	Food safety knowledge, attitude, and hygiene practices of street-cooked food handlers in North Dayi District	Ghana	Cross-sectional	KAP and hygiene practices	None	Poor hygiene despite awareness; vendors need more practical support and infrastructure.	Knowledge-Practice Gap
39.	Nyarugwe et al.	2018	Food safety culture assessment using mixed-methods in emerging economies	Zimbabwe	Mixed-methods	Food safety culture	Food Safety Culture	Revealed gaps between formal safety policies and on-ground practices.	Cultural Implementation Gaps
40.	Karim et al.	2024	Street Food: Hygiene Practices and Knowledge Among Food Hawkers Towards Customer Satisfaction	Malaysia	Survey	Hygiene and customer satisfaction	None	Higher hygiene awareness improves customer satisfaction and loyalty.	Customer-Oriented Hygiene
41.	Bagumire & Rollanda	2017	Hygiene Facilities and Practices for Vended Meats at Highway Markets	Uganda	Observational Study	Meat hygiene practices	None	Lack of infrastructure and training limits food safety among vendors.	Infrastructure & Regulation
42.	Adaku et al.	2024	Barriers to ensuring and sustaining street food safety in a developing economy	Ghana	Mixed-methods	Systemic barriers	None	Regulatory complexity and resource gaps make compliance difficult.	Structural Barriers
43.	Teferi	2020	Street Food Safety, Types and Microbiological Quality in Ethiopia	Ethiopia	Literature Review	Microbial food safety	None	Highlights bacterial contamination in street food due to poor handling.	Microbial Contamination Risk
44.	Sarfo et al.	2025	Factors Influencing the Extent of Food Safety Compliance among Street Food Vendors	Ghana	Empirical	Compliance influencers	None	Social influence, experience, and licensing predict compliance.	Compliance Determinants

45.	Akabanda et al.	2017	Food safety knowledge, attitudes and practices of institutional food-handlers	Ghana	Survey	Food-handler practices	None	Institutional settings also show KAP gaps, relevant for comparison.	Institutional vs. Informal Gap
46.	Ayodele & Panama	2016	Predictors of consumer patronage of street food vendors	Nigeria	Survey	Consumer behaviour	None	Cleanliness and price are key in predicting vendor selection by customers.	Customer Decision Factors
47.	Shiningeni & Mwetulundila	2024	Exploring Ready to Eat Meat Street Vendors' Challenges in Implementing Food Safety	Namibia	Qualitative	Safety implementation challenges	None	Vendors report regulatory ambiguity and limited sanitation facilities.	Implementation Challenges
48.	Van den Biezen, L.	2023	Thriving Amidst Informality-The Development of Vendor Business Schools	Not Specified	Master's Thesis	Vendor development programs	None	Education and mentorship improve vendor performance and safety understanding.	Capacity Building
49.	Santoso, I.	2017	Business Performance of Street Food: The Role of Product Quality, Food Safety and Sanitation	Indonesia	Conference Proceeding	Performance and hygiene	None	Product quality and hygiene positively influence business outcomes.	Business Outcome Linkage
50.	Teangsompong et al.	2024	Consumer trust in Thai street food vendors: Implications for the post-pandemic era	Thailand	Mixed-methods	Consumer trust	None	Cleanliness and presentation remain major factors in post-COVID street food trust.	Post-COVID Consumer Behaviour
51.	Tuffour et al.	2022	Micro-Entrepreneurship: Examining the Factors Affecting the Success of Women Street Food Vendors	Ghana	Survey	Gender and entrepreneurship	None	Women face unique operational barriers but show resilience and adaptability.	Gendered Informal Economy
52.	Reddy, Ricart & Cadman	2020	Driving factors of food safety standards in India: Learning from vendor behavior	India	Case Study	Food safety motivators	None	Vendors comply more when linked to economic incentives.	Motivation-Driven Compliance
53.	Noman, Islam & Islam	2024	Street Food Dynamics in Cox's Bazar: Balancing Economic Vitality with Health Challenges	Bangladesh	Case Study	Balance of safety and livelihood	None	Policy must address both economic and health priorities in informal food systems.	Policy-Economic Balance
54.	Moussa & Yilmaz	2023	Entrepreneurial and Survival Motivations in the Informal Food Sector: A Case Study in N'Djamena, Chad	Chad	Case Study	Motivation and resilience	None	Informality serves as a survival mechanism amid unemployment and lack of formal sector access.	Entrepreneurship as Survival
55.	Lyatuu, P. M.	2025	Coping Strategies and Income of Food Vendors within Bus Stands in Dar es Salaam, Tanzania	Tanzania	Survey	Coping and business strategies	None	Vendors implement informal strategies to cope with harsh operating conditions.	Informal Coping Mechanisms
56.	Mwove, J.	2024	Factors influencing health, food safety and hygiene practices among street food vendors in Meru town, Kenya	Kenya	Cross-sectional	Food safety behavior	None	Strong link between knowledge and compliance; limited by enforcement.	Knowledge-Enforcement Gap
57.	Menes et al.	2019	Street Food: Stories and Insights on Production and Operations, Marketing Strategies, and Vending	Philippines	Mixed-methods	Operations and strategy	None	Vendors use informal networks and adaptive strategies to succeed.	Business Strategy Adaptation
58.	Mbenyane, B. C.	2016	The Role of Business and Government in Shaping South Africa's Food Safety Regime	South Africa	Doctoral Thesis	Food safety regulation history	None	Analyzes regulatory evolution affecting vendor operations.	Policy and Regulation barriers
59.	Soliman et al.	2024	An Integrative Model of Key Factors Determining Consumer Behaviour Towards Street Food	Oman	Survey	Consumer behavior model	Behavioral Model	Trust, hygiene, and presentation predict consumer loyalty.	Consumer Perception Dynamics

60.	Das, S.	2019	A Study on the Business Practices of Street Food Vendors in Guwahati City	India	Survey	Business practices	None	Financial literacy and sanitation awareness vary widely among vendors.	Business Practices Diversity
61.	Kunene, M. L.	2018	Ubuntu Management Strategy in the Informal Sector: A Case of Umlazi Township	South Africa	Case Study	Ubuntu as management philosophy	Ubuntu	Ubuntu fosters trust and collaboration among informal vendors.	Culturally-Informed Governance
62.	Husin et al.	2021	Determinants of Small Business Performance Run by Women Street Vendors in Malaysia	Malaysia	Quantitative Survey	Gender and business performance	None	Marketing, hygiene, and capital access drive performance among women.	Female Vendor Success Factors
63.	Kagaruki et al.	2022	Barriers to the Healthy Plate Model Among Street Food Consumers in Dar es Salaam	Tanzania	Qualitative	Nutritional policy uptake	None	Consumers and vendors lack access and information for healthy food decisions.	Nutrition and Access Barriers
64.	Moges et al.	2024	Sanitary Conditions and Hygienic Practice of Street Food Vendors in Selected Towns of Ethiopia	Ethiopia	Cross-sectional	Hygiene and health risks	None	Poor sanitation and regulatory gaps threaten public health.	Sanitation Infrastructure Gap
65.	Nakpathom et al.	2023	Customer Perception Toward Street Food Hygiene During the Pandemic: Case of Bangsean, Thailand	Thailand	Survey	COVID-19 impact on perception	None	Pandemic increased customer sensitivity to hygiene standards.	Pandemic-Induced Behavior Shift
66.	Apandi et al.	2024	Impact of Hygiene Practices by Street Stall Hawkers on Customer Repurchase Intention	Malaysia	Quantitative Survey	Hygiene and consumer loyalty	None	Clean practices directly boost return customer behavior.	Hygiene and Loyalty Link
67.	Okumus & Sonmez	2018	An Analysis on Current Food Regulations for and Inspection Challenges of Street Food: Case of Florida	USA	Case Study	Regulatory inspection challenges	None	Highlights the complexity of enforcing street food regulations.	Policy and Regulation barriers
68.	Daudzai, M. H.	2021	Economics of Street Food Vendors in Nigeria and Afghanistan	Nigeria, Afghanista n	Master's Thesis	Comparative economics of vending	None	Informal vendors face structural economic barriers and high risk.	Economic Vulnerability
69.	Abd Rahman et al.	2023	The Influence Of Penang's Street Food On Customers' Intention To Return	Malaysia	Survey	Consumer loyalty and food culture	None	Cultural familiarity and hygiene increase intention to repurchase.	Cultural Affinity and Return
70.	Maphisa, S.	2020	Policy Implementation Gaps in Urban Food Informality: The Case of Durban	South Africa	Policy Analysis	Implementation failures	None	Municipal policies lack inclusive enforcement practices for informal food vendors.	Governance Gap
71.	Hossain, M. & Huda, F.	2024	Behavioral Insights into Hygiene Practices Among Urban Vendors in Dhaka	Banglades h	Behavioural Study	Hygiene behaviour	HBM	Behavioural framing improves compliance.	Behavioural Intervention Efficacy
72.	Ayalew, G.	2018	Food Safety and Regulatory Practices in Addis Ababa	Ethiopia	Case Study	Urban food safety	None	Market-level enforcement is limited by resource and training gaps.	Institutional Weakness
73.	Chauke, M.	2023	Access to Water and Sanitation for Informal Vendors in Gauteng	South Africa	Qualitative Study	Water access	None	Infrastructure gaps inhibit safe food preparation.	Water-Sanitation Link
74.	Abdurrahman, A.	2024	Mobile Technology Use Among Urban Street Food Vendors in Lagos	Nigeria	Field Survey	Technology and food safety	BSC	Mobile apps improved vendor visibility and hygiene tracking.	Tech-Enabled Safety Monitoring
75.	Phiri, T. & Banda, K.	2023	Conflict Between Local Governments and Vendors Over Space	Zambia	Case Study	Spatial policy conflict	None	Vendors are regularly displaced despite permits, affecting their livelihood.	Spatial Exclusion

76.	Osei, B., & Boateng, J.	2023	Pricing, Competition, and Quality Control Among Urban Street Food Vendors	Ghana	Economic Study	Market dynamics	None	Quality and safety are often compromised under price competition.	Quality vs. Price Tension
